# Climate Change May Boost the Invasion of the Asian Needle Ant

**DOI:** 10.1371/journal.pone.0075438

**Published:** 2013-10-04

**Authors:** Cleo Bertelsmeier, Benoît Guénard, Franck Courchamp

**Affiliations:** 1 Ecologie, Systématique & Evolution, Univ. Paris Sud, Orsay, France; 2 Biodiversity and Biocomplexity Unit, Okinawa Institute of Science and Technology, Okinawa, Japan; Stanford University, United States of America

## Abstract

Following its introduction from Asia to the USA, the Asian needle ant (*Pachycondyla chinensis*) is rapidly spreading into a wide range of habitats with great negative ecological affects. In addition, the species is a concern for human health because of its powerful, sometimes deadly, sting. Here, we assessed the potential of *P. chinensis* to spread further and to invade entirely new regions. We used species distribution models to assess suitable areas under current climatic conditions and in 2020, 2050 and 2080. With a consensus model, combining five different modelling techniques, three Global Circulation (climatic) Models and two CO_2_ emission scenarios, we generated world maps with suitable climatic conditions. Our models suggest that the species currently has a far greater potential distribution than its current exotic range, including large parts of the world landmass, including Northeast America, Southeast Asia and Southeast America. Climate change is predicted to greatly exacerbate the risk of *P. chinensis* invasion by increasing the suitable landmass by 64.9% worldwide, with large increases in Europe (+210.1%), Oceania (+75.1%), North America (+74.9%) and Asia (+62.7%). The results of our study suggest *P. chinensis* deserves increased attention, especially in the light of on-going climate change.

## Introduction

Among the over 12,000 described species of ants [1], more than 200 species have established populations outside their native range [2]. The rate of new species introductions continues to increase due to the ever growing human-mediated transportation via international trade and tourism [3]. Only a small subset of introduced ant species eventually becomes invasive, but these species can have a large impacts [4]–[6]. They can cause significant biodiversity losses, in particular as extremely efficient predators and competitors [7]. For example, most native ant species may be eliminated from the invaded habitat and a variety of other taxa, ranging from soil microbes to small mammals, can be negatively affected [6], [8]. In addition, invasive ants can disturb ecological networks, such as seed dispersal mutualism, thereby impairing ecosystem functioning [7]. Finally, they often damage agroecosystems and are a nuisance to humans by infesting estates, leading to high economic costs [9].

Ants are known to be very sensitive to changes in temperature and humidity, because it affects their survival [10], foraging activity [11] and foraging networks [12] and dominance hierarchies [13]. It is generally accepted that with climate change, many invasive ant species will progressively colonize higher latitudes and altitudes, where the currently too cold climatic conditions are expected to become more suitable [14], [15]. In this regard, several studies have used species distribution models [16]–[19] or physiological experiments [11], [20], [21] to investigate the relationship between temperature and humidity and ant distribution. Climate is one of the most important factors influencing the distribution of ants [22]–[24] and climatic suitability has been shown to be even the most important factor responsible for the current global distribution of the invasive Argentine ant, *Linepithema humile* [25]. Climate can therefore serve as an important proxy to estimate the potential distribution of invasive ants worldwide. It is generally recognized that climate change is going to be a major determinant of species physiology, phenology and range shifts during this century [26]. However, few studies have gone beyond the estimation of current potential invasive range and forecast also the future potential distributions of invasive species in general (but see [27]–[29]). Furthermore, such pioneer studies estimating the future potential ranges of invasive ants have concentrated on the few species of *Linepithema humile* [25], [30] *Solenopsis invicta* [31] and *Pheidole megacephala* [16], leaving a great knowledge gap for most other major invasive ant species.

Risk assessments conducted prior to the arrival of an invasive species are a vital component of biosecurity preparedness, because most species, including invasive ants, are extremely difficult to eradicate once they become established [32]. Providing a spatial model of relative climatic suitability is an important component of risk assessments to prioritize surveillance efforts for invasive species with a high likelihood of establishing in a particular region. The greater the extent of an invasion, the higher the environmental impacts of management attempts and the difficulty of achieving successful eradication [33]. It is known that reactive programs have generally a higher cost than proactive programs [34]. In this context, the Asian needle ant, *Pachycondyla chinensis*, is of utmost interest. Despite its introduction from Asia to the eastern part of North America in the first part of the 20^th^ century, the invasion of this fast-spreading species was only detected recently in a wide range of habitats in North America, including mature temperate forests, where it causes a strong decline in native ant abundance [35]. In addition, the species has been shown to disrupt an ant-seed dispersal mutualism by displacing a native keystone ant species [36]. The species’ negative impact on native seed dispersers has been compared to the impact of Argentine ant [36], which is among the “100 of the worst invasive species” list of the IUCN [37] and has enormous impacts on biodiversity [38]. Additionally, *P. chinensis* is a growing concern for public health due to its powerful, and sometimes deadly, sting [35].

Consequently, there is a strong need to develop predictive models of the potential distribution of this highly invasive species, both currently and in the future with predictions of climate change. Here we use species distribution models to: (1) to quantify the current potential distribution worldwide and within six broad geographic regions; and (2) quantify the change in potential distribution with global climate change at the global and regional levels.

## Materials and Methods

### Species Distribution Data

Species distribution models search for a non-random association between environmental predictors and species occurrence data to make spatial predictions of potential distribution. Because our models should include the full set of climatic conditions under which the target species can exist, we included occurrence points (presence only data) from both invaded and native habitats [39]. In total, we used 283 occurrence points, 219 from North America (invaded range) and 64 from Asia (native range) (Fig. S1). The exact distribution of *P. chinensis*’ native range is problematic as this species belongs to a large and taxonomically unresolved complex of species [40]. To maximize data integrity, the data used for modeling were limited to specimens collected in its native range and identified by one of the authors (BG) and specimens strictly identified as *P. chinensis* in literature [40], [41]. In its introduced range, where *P. chinensis* identification is not problematic, localities were extracted from literature, museum records and personal collecting (BG).

For models requiring absence data, 10,000 pseudo-absence (background) points were generated randomly from all around the world to provide background data. This is a classic procedure because confirmed absence data is difficult to obtain for most species and requires great sampling efforts [42]. True absence data might improve the model accuracy because some pseudo-absence points may be drawn from regions where the species is actually present, but has not been recorded. However, it is not possible to base our projections on true absences due to lacking large-scale absence data of the species. In addition, in the case of invasive species, even a true absence point may indicate a suitable location that the species has not yet been introduced to due to a lack of opportunity. Therefore, we believe that pseudo-absence data can serve as a reasonable proxy.

### Climatic Predictors

Climatic predictor data was sourced from the Worldclim dataset. The 19 Worldclim variables represent annual trends (e.g. mean annual temperature, annual precipitation) and extreme limiting environmental factors (e.g. temperature of the coldest and warmest months, precipitation of the wettest or driest quarter) and are known to influence species distributions [45], [46]. All Worldclim variables are 30-year averages of monthly temperature and rainfall values from 1960–1990 [43], which is characteristic of the climate that the species experienced when the occurrence point was collected or the species established in this locality. We modelled the species niche based on 4 of the 19 bioclimatic variables that were not collinear (pair-wise r_Pearson_ <0.75). The selected variables were (in the order of their relative contribution to the Maxent model): Precipitation of the driest month, isothermality, precipitation of the warmest quarter and maximum temperature of the warmest month. These variables are believed to directly influence ant distributions because many features of ant biology are sensitive to small differences in temperature [16] or humidity, for example foraging [11], oviposition rates [44], survival [10], the structure of foraging networks [12].

Future climatic data were sourced from the 4^th^ IPCC assessment report [47]. The direct output of Global Climate Models is provided in the form of very large (500 km) grid cells because of the heavy calculations needed for the simulation of geophysical processes. To get a better resolution required for species distribution modelling, climate centres use statistical models to infer climatic variation at a more local scale, “downscaling” the data by using the WorldClim data for ‘current’ conditions for calibration. Therefore the projections at different time horizons can be compared. In order to consider a range of possible future climates, we used downscaled climate data from three Global Circulation Models (GCMs), provided by different climate centres, each based on different geophysical assumptions: the CCCMA-GCM2 model; the CSIRO-MK2 model; and the HCCPR-HADCM3 model [47]. We also used two extreme Special Report on CO_2_ Emission Scenarios (SRES): the optimistic B2a and pessimistic A2a scenarios. In total, we used six future climatic scenarios (3 GCM×2 SRES). Data for the future climatic projections were climate data averaged across a decade, centred on the focal year (e.g. 2020) [47].

Worldclim data is the standard source of climate data for species distribution models. However it is poor at interpolating climate in topographically complex regions such as mountains or coastal regions [48]. But the focus of our study is projections at the global scale with a spatial resolution of 10 arcmin (approx. 18.5×18.5 km pixel), where complex coastlines are not visible. Predictions based on coarser resolutions are more likely to be controlled by climatic predictors, whereas fine-scale, patchy distributions at a smaller scale are more likely to be determined by micro-topographic variations or habitat fragmentation [49].

### Species Distribution Modelling

In order to make spatial predictions of potential distribution, we used species distribution models (SDMs), which explain the species’ current distribution based on a set of climatic predictor variables. It has been shown that model outputs are sensitive to the algorithms, climatic data from different global climate models and different human development scenarios [50]. One way to deal with these uncertainties in species distribution modelling is to conduct a consensus forecast which can be defined as combining multiple simulations across a range of possible initial conditions and different classes of models [51].

To generate the consensus forecasts we used five machine learning methods, which are a set of algorithms that learn the mapping function or classification rule inductively from the input data [52]. The first two models were based on one- and two- class Support Vector Machines (SVMs) [39], [31]. Two-class SVMs (SVM2) seek to find a hyperplane that maximally separates the two target classes. Recently, one-class SVMs have also been developed [42] that distinguish one specific category from all other categories. Third, we used Artificial Neural Networks (ANN), which extract linear combinations of the input variables as derived features (synthetic variables), and model the output as a nonlinear function of these derived features [42], [54]. Fourth, we used Classification Trees (CT), which partition the response variable into increasingly pure binary subsets with splits and stop criteria [41], [42], [53]. Finally we used the Maximum Entropy Method (Maxent) which estimates a probability distribution of a species being present by seeking the most widespread distribution, given a set of constraints [18], [57], [58]. For a more detailed description of these algorithms see [16], [19]. All models were run using the ModEco Platform with default parameters [59].

A clear limitation of modelling is that outputs are dependent on the specifically chosen input settings, in this instance the algorithms, global climate models and scenarios of human development. To minimise potential resulting variation, we conducted consensus forecasts [51] using the outputs of the five different modelling techniques detailed above with each of three climate models (GCMs) and two CO_2_ emission scenarios (SRES). The purpose of consensus forecasts is to separate the signal from the “noise” associated with the errors and uncertainties of individual models, by superposing the maps based on individual model outputs. Areas where these individual maps overlap are defined as areas of “consensual prediction” [51]. This is different from averaging the individual projections, as the area predicted by the consensus forecast can be smaller than any individual forecast if there is little spatial agreement (*i.e.,* overlap) between individual forecasts. Simple averaging across individual forecasts is considered unlikely to match reality [51].

The contribution of the individual models (i.e. the spatial prediction of “suitable range”) was weighted according to their AUC (section on model validation) in order to enhance contribution of models with higher model performance values (see [18]). Only binary projections (present or absent) have been combined to generate the consensus model because continuous outputs can have different meanings for different models and cannot be simply added together [59]. The combination of the individual forecasts then yields a projection (the consensus model), where the value of pixels vary between 0 and 1 and can be interpreted as a probability of the species occurring in each pixel [51].

The consensus model was generated using all 30 individual projections, each based on a different combination of CO_2_ scenario×GCM×modelling technique, yielding a consensus projection for 4 time horizons (current, 2020, 2050 and 2080). The future climatic projections that we used as a basis of our models are in fact averaged climate data across a decade, centred on the focal year (e.g. 2020) [47].

### Model Validation

Model robustness was evaluated using the AUC of the ROC curve, which is a nonparametric threshold-independent measure of accuracy commonly used to evaluate species distribution models (e.g., [18], [60]). We used the AUC because it does not depend on the selected classification threshold, and it readily indicates if a model discriminates correctly between presence and absence points [18], [60]. AUC values range from 0 to 1, where a value of 0.5 can be interpreted as a random prediction. AUC between 0.5 and 0.7 are considered low (poor model performance), 0.7–0.9 moderate and >0.9 high ([42] and references therein). For model evaluation, the data needs to be split into a train and a test group. Here, we used 10-fold cross-validation, whereby the data was split into 10 equal parts, with 9/10 of the observations used to build the models and the remaining 1/10 used to estimate performance. Validation was repeated ten times and the estimated performance measures were averaged [42], [61].

### Assessing Climatically Suitable Areas

Studies with *a priori* objectives may use a range of different threshold values [62] to determine habitat suitability. As this was not the case here, we applied a limit whereby pixels with a probability of presence exceeding 0.5 were classified as “suitable” area, as is frequently done for binary classification for species distribution modelling [42], [63]. Users of our models may want to minimize the chance of either over- or under-prediction of potential distribution (omission or commission errors) and to apply a different threshold. For example, for management decisions it could be better to apply a more “prudent” (lower) threshold that lowers the probability of omission errors. To allow these user-specific applications of our models, we provide maps with a continuous output with a probability of presence between 0 and 1 (with 0.1 intervals).

In addition, we created a difference map (future suitability map – current suitability map), which showed relative differences that are independent of any classification threshold and indicated areas where the climatic suitability improved or decreased. Second, we generated a “shift” map where we mapped the net gains, losses and stable ranges under current and future climatic conditions. Third, we calculated two indices that have been recently proposed as complementary measures to evaluate the extent of spatial shift [64]: the spatial congruence index (2a/2a+b+c), based on the Sorensen-Dice dissimilarity measure, and the stable range (a/(a+b)), which is a measure of spatial shift vs stability, whereby a = area suitable currently and in the future, b = area suitable currently only, and c = area suitable in the future only. Spatial analyses were carried out using DIVA-GIS [65] and Arcgis v. 9.3.

## Results

The AUC values indicated excellent model performance for all five algorithms in predicting the species’ distribution based on the consensus model (AUC values: SVM1 = 0.968, SVM2 = 0.991, Maxent = 0.998, ANN = 0.991, CT = 0.997).

### Current Climatic Conditions

**Figure 1:**
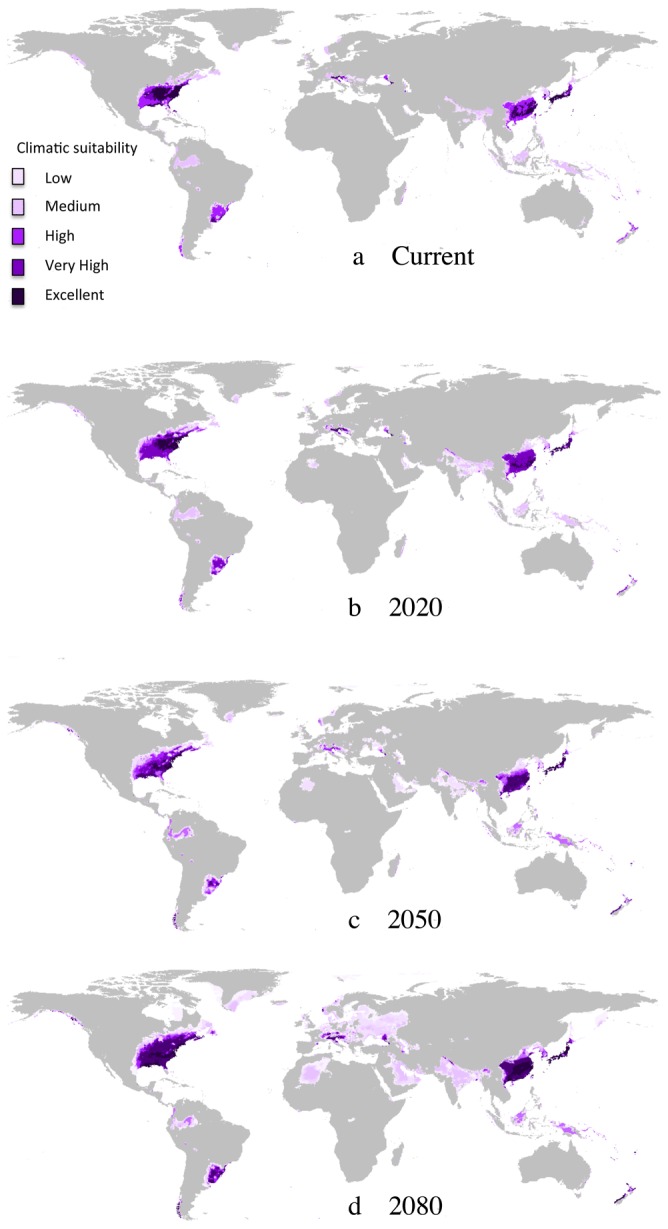
Maps of potential distribution. Climatic suitability ranges from “low” (light purple) to “excellent” (dark purple). (**a**) Current climatic conditions (**b**) Consensus model of 6 future climatic scenarios (3 GCM×2 SRES) for 2020, (**c**) 2050 and (**d**) 2080.

**Figure 2:**
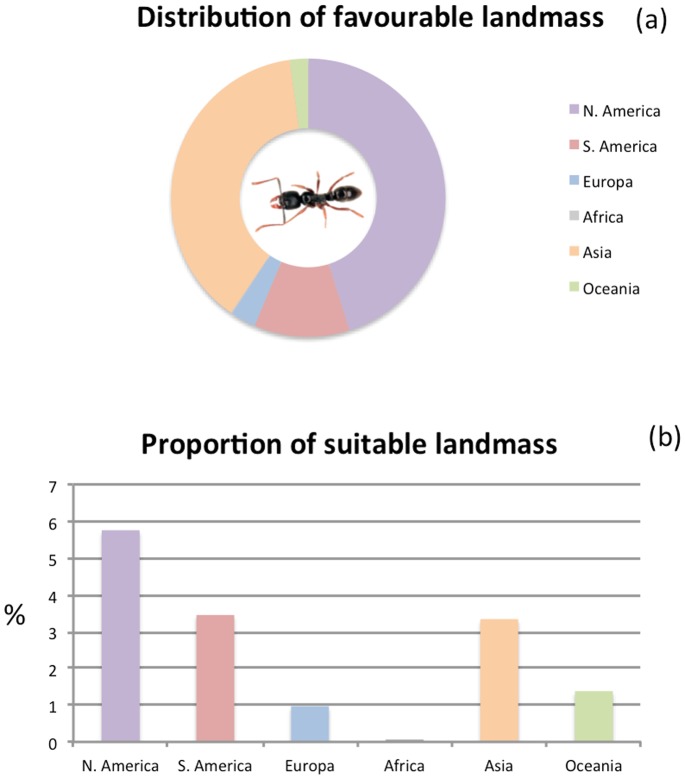
a+b Distribution and proportion of suitable landmass under current climatic conditions among six biogeographic regions.

Maps of the consensus model under current and future climatic conditions by 2020, 2050 and 2080 indicated large and increasing suitable areas for *P. chinensis* (Fig. 1a–d). Under current climatic conditions, 3.33% of global landmass was predicted to be suitable for *P. chinensis*. The suitable range was unequally distributed among biogeographic regions, with the highest relative amount of suitable landmass found in North America (45%), followed by Asia (38%), South America (11%), Europe (3%) and Oceania (2%) (Fig. 2a). The relative proportion of suitable landmass was also highest in North America (Fig. 2b).

### Climate Change Impacts

**Figure 3:**
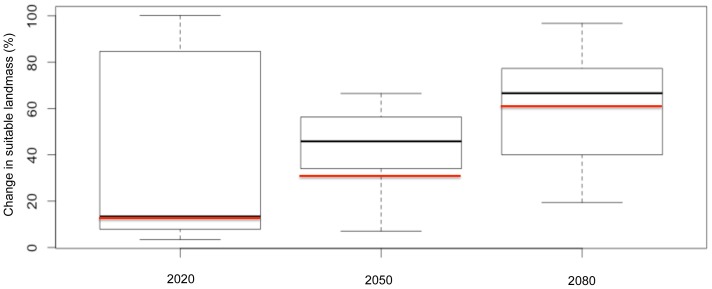
Change in suitable landmass over time relative to the currently suitable landmass. The boxplot represents variation of projections across six future climatic scenarios, each based on a different combination of Global Climate Model×CO_2_ emission scenario, per time horizon (± s.d). The red line indicates the value of the consensus model.

The suitable range for *P. chinensis* increased dramatically with projected climate change. In 2020 the potential range increased by 15.6%, in 2050 by 29.3% and in 2080 this increase reached +64.9% of the currently suitable landmass (Fig. 3).

By 2080, changes in suitable landmass differed greatly among biogeographic regions, with large increases in Europe (+210.1%, *i.e*., +363,117 km^2^), Oceania (+75.1%, *i.e*., +94,332 km^2^), North America (+74.9%, *i.e*., +1,972,781 km^2^) and Asia (+62.7%, *i.e*., +1,403,693 km^2^) and a decrease in Africa (−22.9%, *i.e*., −2,042 km^2^).

### Spatial Shifts of Suitable Conditions

**Figure 4:**
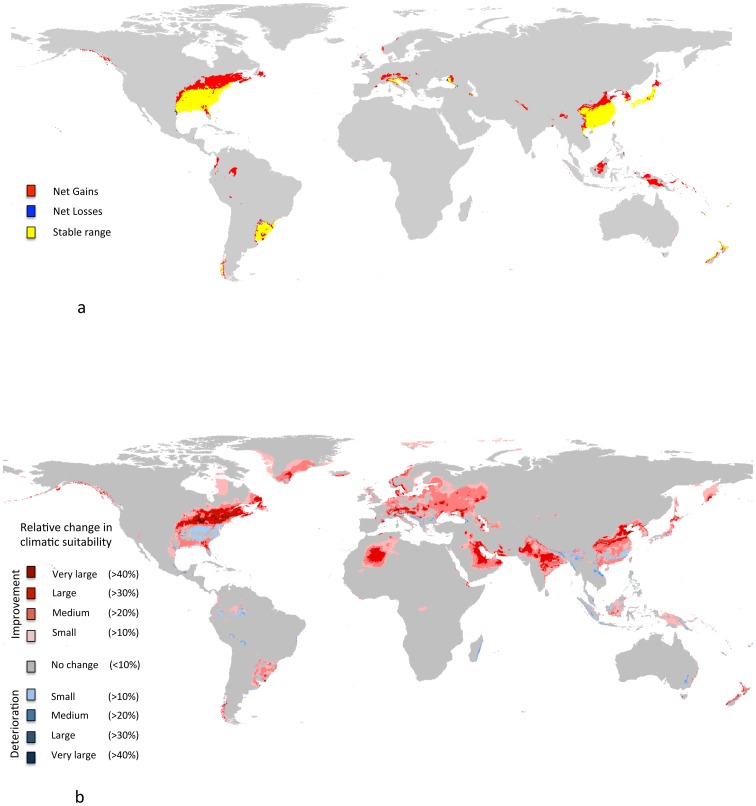
Differences in suitable climatic conditions between the current climate and the consensus projection in 2080. (a) Net gains (red), losses (blue) and stable range (yellow), (b) Relative differences in suitability : red colors indicate improved climatic suitability (light to dark red gradient indicates the relative change in suitability), blue colors indicate deteriorated climatic suitability for *P. chinensis.*

The net changes in suitable landmass were almost exclusively due to gains in potential distribution (+4,350,682 km^2^, Fig. 4a). Very small areas of suitable landmass was lost (i.e., suitable under current but not under future climatic conditions: −88,300 km^2^, Fig. 4a). These increases were from range expansions at the edge of the suitable range, but also entirely of new areas that became suitable (e.g. Europe, Northern Brazil or Indonesia, Fig. 4a). The stable range index was 0.569, meaning that only 56.9% of *P. chinensis*’ current potential range will remain suitable in the future. Spatial congruence was 0.722, which provides a measure of the stability (stable range) vs shift (losses and gains), indicating that 72.2% of all current and future suitable areas can be considered as “stable range” over time, whereas 27.8% will be either suitable currently or in the future, but not under both climatic scenarios.

The relative differences between the current and future areas of suitability, indicated that *P. chinensis* was likely to experience higher relative climatic suitability in all biogeographic regions, including large parts of north Africa, Arabia, India, South East Asia, north east America and eastern Europe (Fig. 4b). Few areas, such as the eastern USA showed a slight decrease of suitability (areas in light blue, Fig. 4b).

## Discussion

The Asian needle ant is native to Asia and has already invaded the South Eastern part of the USA [35]. Our models suggest that the species has a far greater invasive potential and is capable of invading large parts of the global landmass on several continents, particularly north east America, South East Asia and south east America. In addition the consensus models suggest that climate change will exacerbate the risk of invasion by *P. chinensis* globally. Strikingly, the global suitable landmass is predicted to increase by 64.9% with climate change. At the regional scale, most of *P. chinensis*’ potential distribution was found in its Asian native range and in North America where the species is now considered invasive [35]. With climate change, the amount of suitable area for *P. chinensis* is predicted to greatly increase based on more suitable conditions, by 74.9% in North America. In addition, our models also predict a potential expansion into new biogeographic regions that should become suitable by 2080, in particular Europe, South America or Indonesia and an expansion in Asia relative to its current known distribution range.

In addition to direct climatic suitability, climate change could enhance the invasion likelihood by disadvantaging other competing ants in the invaded areas. Ant community structures are known to be temperature-dependent [13], [66], and therefore a change in temperature might lead to new invaders dominating. For example, *P. chinensis* has recently been found to establish in sites dominated by the invasive Argentine ant, *L. humile*, resulting in a dominance swap and even localised extinction of *L. humile* [65]. Because *P. chinensis* does not seem to be behaviourally dominant, this dominance shift has been attributed to differences in the climatic preferences of the two species, with *P. chinensis* establishing nests and expanding is population earlier in the season when temperatures were lower before *L. humile* populations could expand [65].

Our consensus models for the potential distribution had a high accuracy (good to excellent AUC) and were designed to include a broad range of climate change scenarios, with an optimistic B2a and a pessimistic A2a CO_2_ emission scenario and three global circulation climatic models [47]. We additionally reduced uncertainty due to single modelling methods by building models with five different algorithms that contributed to the final consensus forecast [51]. Nevertheless, inherent uncertainty in the spatial projection remains because of the underlying assumptions shared by all species distributions models [49], [46] in that they assume that the species is in equilibrium with its environment and therefore its current distribution reflects the ideal climatic conditions for the species, which can be used to model its potential distribution. That means that a model of the potential distribution of an invasive species with climate change has to make two extrapolations: 1) in space (invasion of a different place); and 2) in time (with future climate change). However, niche shifts during invasions are possible and have already been observed [67]–[69] and species may display phenotypic plasticity or show evolutionary adaptations, such as has been shown for the Asian ladybeetle which has the same invasion pattern as *P. chinensis*, dispersing from Asia to the east coast of North America [70]. Therefore, projections for invasions under climate change come necessarily with some uncertainty and should be only viewed as an attempt to evaluate future trends and invasion risks, and not as a precise prediction at a small scale. For example, a new occurrence point of *P. chinensis* has been recently recorded in Washington D.C. by the School of Ants project [71], where the species does not find ‘excellent’ climatic conditions according to our projections. One of the inherent problems of species distribution models is that the species may be able to occur within areas predicted to be of relatively low suitability, if the microclimatic conditions favour the species or if it is associated to human infrastructure. Despite these limitations, species distribution models are generally considered to deliver useful approximations [72], [73].

A further factor to consider is that numerous native species are predicted to suffer the effects from climate change [26]. This may increase the vulnerability of the community by decreasing the biotic resistance to new invaders [74], [75]. This could even further exacerbate the invasion risk of *P. chinensis*.

Our study solely focused on the role of climate change on the potential distribution of a newly invasive ant species because climate has been shown to be very important in determining the distribution of ants at a global scale [22]–[24] as well as being the most important factor influencing the global invasion of the Argentine ant [25]. Therefore, climate is probably a crucial factor limiting the distribution of other invasive ants, such as *P. chinensis*. For future studies, predictions of invasive potential will be advanced by investigations into the influence of other abiotic factors and drivers of species displacement. At a finer scale for example, topographic or terrain variables, such as elevation, geomorphology or hydrology can be important [42]. For example, appropriate soil moisture levels can be an important requirement for nest location of ants. Also important is the effect of disturbance regimes, because invasive ants are frequently associated with disturbed habitats [76], [77]. However, it should be noted that *P. chinensis* has been found to invade intact forests [36].

Our results support the view that biological invasions could increase due to climate change [60], [61], [62], [10], [11] and show it can do so dramatically. In this way two of the most important threats to global biodiversity (invasive species and climate change) may interact synergistically. An important observation is that the potential distribution of *P. chinensis* exists at a wide range of latitudes, and thus this species’ potential range did not simply shift to higher latitudes. Consequently, invasion risk was exacerbated globally with entirely new areas covering large amounts of landmass becoming suitable. Given the important ecological impacts of *P. chinensis* [35] and its ability to potentially even displace one of the most aggressive and dominant species of invasive ants, the *L. humile* [81], clearly it is important that surveillance efforts of this species are increased to prevent further spread and aid early detection. Eradication of well established large invasive ant populations can be extremely challenging [32] if not impossible, but early detection of small incipient populations can enable managers to carry out early responses and achieve eradication [34]. The use of species distribution models to inform risk assessments is increasingly becoming standard, but a spatial model should be always viewed in the light of the many uncertainties associated with the approach [58]. Species distribution models can serve as a guide to prioritize surveillance efforts in certain regions. Ideally, this approach should be complemented with interception data at ports of entry. The results of our study suggest *P. chinensis* deserves increased attention in new regions of the world (e.g., Europe and South America) as a rising exotic species of significance.

## Supporting Information

Figure S1 **Occurrence points of** ***P. chinensis***
(TIF)
