# Supplementary figures and images for: Climate Change May Boost the Invasion of the Asian Needle Ant
Source: PLoS One. 2013 Oct 4;8(10):e75438. doi: 10.1371/journal.pone.0075438 (PMC3790791; doi:10.1371/journal.pone.0075438)

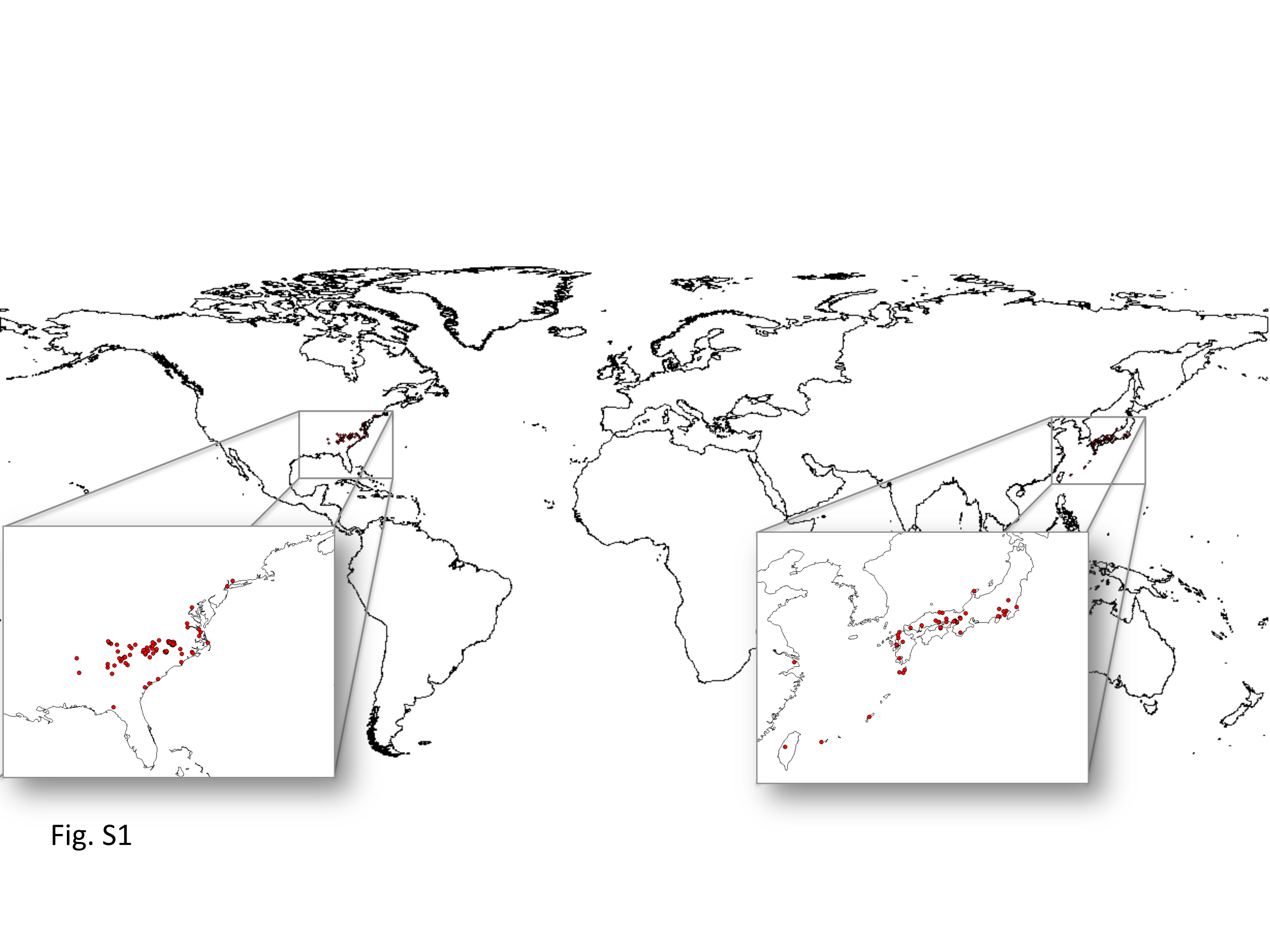

Supplement: Figure S1 — Occurrence points of P. chinensis (TIF) [file pone.0075438.s001.tif]
